# HIV Care for Adolescents and Young Adults: A Cross-Sectional Comparison of Nurse Practitioner and Physician Roles in Engagement in Care, Trust, and Clinical Outcomes

**DOI:** 10.1177/23259582261485418

**Published:** 2026-10-01

**Authors:** Emily Anne Barr, Suzanne E. Courtwright, Sheryl Malone-Thomas, Lori Silveira, Deborah Kacanek, Paul Cook, Sean M. Reed, Hillary Dunlevy

**Affiliations:** 1 College of Nursing, 631580 University of Colorado, Aurora, CO, USA; 2 Cizik School of Nursing, 16171 University of Texas Health Science Center at Houston, Houston, TX, USA; 3 16074 Department of Research, University at Buffalo School of Nursing, Buffalo, NY, USA; 4 15760 Center for Healthcare Delivery Research & Innovation, Columbia University School of Nursing, New York, NY, USA; 5 Department of Pediatrics, University of Colorado School of Medicine, Aurora, CO, USA; 6 1857 Center for Biostatistics in AIDS Research, Harvard T.H. Chan School of Public Health, Boston, MA, USA; 7 Department of Medicine, 129263 University of Colorado School of Medicine, Aurora, CO, USA

**Keywords:** HIV infections/prevention & control, nurse practitioners, adolescent health services, young adult, patient-provider trust, telehealth, health workforce, patient-reported outcomes

## Abstract

**Background:**

Nurse practitioners (NPs) may help address HIV workforce shortages, but evidence comparing health outcomes in youth remains limited. This study compared clinical and patient-reported outcomes among adolescents and young adults with HIV (AYA-WH) receiving NP versus physician care.

**Methods:**

In this cross-sectional study, health record and survey data from AYA-WH (N=109) receiving care in Colorado, USA (June 2019–November 2020) were analyzed. Outcomes included viral suppression, care engagement, telehealth use, trust, adherence, and HIV stigma.

**Results:**

Viral suppression and engagement in care were comparable between NP and physician groups. Youth receiving NP care had more visits, greater telehealth use, higher CD4 counts, and were more likely to report higher patient-provider trust. Adherence and stigma did not differ by provider type.

**Conclusions:**

NPs are well-positioned to expand access to youth-focused HIV care while maintaining outcomes comparable to physician providers.

## Plain Language Summary

Many healthcare providers who specialize in HIV care are approaching retirement, creating concerns about future access to HIV services. Finding ways to expand the HIV workforce is especially important for adolescents and young adults, who often face challenges staying engaged in care and have lower rates of HIV testing and diagnosis than other age groups. Nurse practitioners (NPs) are advanced practice nurses who provide a wide range of healthcare services, including HIV prevention and treatment. However, little research has examined whether HIV care provided by NPs is comparable to care provided by physicians for young people living with HIV. This study included 109 adolescents and young adults receiving HIV care in Colorado. We compared health outcomes, healthcare use, and patient experiences among those receiving care from NPs and those receiving care from physicians. We found that young people receiving care from NPs and physicians had similar rates of viral suppression, meaning the virus was well controlled in both groups. Engagement in care, medication adherence, and experiences of HIV-related stigma were also similar. Participants receiving care from NPs had more healthcare visits, used telehealth more often, and were more likely to report higher levels of trust in their healthcare provider. They also had higher average CD4 counts, a measure of immune system health. These findings suggest that NPs can provide high-quality HIV care for adolescents and young adults that is comparable to physician care. Expanding opportunities for NPs to deliver HIV care may help address workforce shortages, improve access to services, and support the long-term health of young people living with HIV.

## Implications and Contribution Statement

This study demonstrates that nurse practitioners provide HIV care to adolescents and young adults that is comparable to physician care across key clinical outcomes, including viral suppression and engagement in care. Participants receiving NP care had more frequent visits, greater telehealth utilization, and were more likely to have HCR Trust Scale-R scores at or above the sample median. These findings support the inclusion of NPs in efforts to expand the HIV clinical workforce and improve access to youth-focused HIV services. The study contributes novel evidence to inform workforce planning and highlights the potential importance of trust in youth-focused HIV care.

## Introduction

A shortage in the U.S. HIV clinical provider workforce limits equitable access to HIV prevention and treatment services, particularly for adolescents and young adults aged 13–34, who account for 58% of HIV infections nationwide.^1-4^ This workforce shortage is a structural determinant of health that, similar to other structural determinants including geography, insurance status, racism, and migration, is associated with inequitable health outcomes for people with HIV and threatens progress to end the HIV epidemic (EHE).^2,5-8^ Importantly, experts agree that achieving the EHE goals is dependent on addressing HIV workforce limitations and improving capacity to provide HIV prevention and treatment services.^8^

Predicted more than a decade ago, the HIV workforce shortage reflects the retirement of early HIV specialists, limited growth in infectious diseases training programs, and insufficient HIV-focused pathways in primary care residencies.^9-13^ Despite efforts to expand training, infectious disease fellowship enrollment declined by 8.1% between 2008 and 2022, and more than half of fellowship programs did not fill available positions in 2024.^4^ Further, graduates of dedicated HIV training pathways within family medicine residency programs represented only 38% of physicians providing HIV health services.^9^ Compounding these challenges, few HIV-trained clinicians practice in southern or rural regions, which now represent the epicenter of the U.S. HIV epidemic.^3,14^

National workforce projections further underscore these inequities. Health Resources and Services Administration analyses estimate that the rural infectious disease workforce will meet only 14% of projected demand between 2024 and 2036.^15^ Similarly, the adolescent medicine workforce, another critical pipeline for youth HIV prevention and treatment, is projected to decline and remains unevenly distributed geographically, including lower access in the U.S. South.^16^ Insurance access further constrains care delivery, as an estimated 25% of family physicians and 15% of pediatric primary care physicians caring for youth with HIV no longer accept Medicaid, the primary payor for people living with HIV.^17^ Together, these factors create persistent barriers to youth-focused HIV prevention and treatment and threaten progress for the Ending the HIV Epidemic (EHE) initiative, which aims to reduce new HIV infections in the United States by 2030.^1,18^

These workforce gaps are particularly concerning given that youth aged 13–24 have the highest proportion of undiagnosed HIV infections at 44%, substantially exceeding other age groups.^19^ HIV testing rates in youth with HIV (YWH) remain low, with fewer than one in four youth reporting having been tested.^20,21^ Evidence indicates that youth are significantly more likely to undergo HIV testing when they have clinicians with whom they can discuss sexual health and HIV openly, underscoring the importance of accessible, developmentally responsive care.^20,21^ Ensuring a workforce capable of engaging youth in these conversations is therefore central to HIV prevention and early treatment efforts. In addition to workforce shortages, youth with HIV face multiple structural barriers that affect engagement in care (EIC) and health outcomes. Challenges related to insurance coverage, transportation, housing instability, food insecurity, and access to HIV-specialized providers have been associated with HIV care attendance, antiretroviral therapy use, and viral suppression among adolescents and young adults with HIV.^7,22^ These structural determinants highlight the importance of accessible, youth-centered HIV care models and a workforce equipped to address both clinical and social needs.

Nurse practitioners (NPs) represent a rapidly expanding segment of the U.S. healthcare workforce and are well positioned to address these gaps.^23,24^ As nationally board-certified advanced practice nurses, NPs receive graduate-level training in advanced assessment, pharmacology, and population-focused care, with an emphasis on addressing social, behavioral, and structural determinants of health. Federal investments in HIV-focused NP training programs, including those supported by HRSA, have further strengthened this workforce.^25^ Prior studies demonstrate that adult HIV care delivered by NPs achieves clinical outcomes comparable to physician-led care, with some evidence of advantages in prevention counseling, adherence support, and retention.^26,27^

However, the evidence base specific to HIV care for YWH delivered by NPs remains limited. Although prior studies have compared NP and physician HIV care in adult populations, little is known about NP-delivered HIV care among adolescents and young adults with HIV. To our knowledge, no study has examined HIV-specific clinical outcomes, patient-provider trust, and telehealth utilization together in a youth-focused HIV population. Importantly, relational aspects of care, including trust, may be especially salient for sustaining engagement in this population. Given the disproportionate burden of HIV disease and elevated risk of disengagement from care among YWH, this gap in knowledge can limit efforts to improve equitable access to HIV care for this vulnerable population and ultimately make progress toward the goals of the Ending HIV Epidemic initiative.^1,28^

This study was conceptually informed by the Trust-Link Relational Transition Model,^29^ which frames trust as a relational process that shapes engagement, continuity, and health outcomes for youth with chronic illness. The model integrates nursing and trust theory to emphasize informed trust, relational presence, and collaborative care as mechanisms that support youth engagement during vulnerable care transitions. Given evidence that trust mediates retention and adherence in HIV care, particularly among youth facing stigma and developmental transitions, this framework guided our selection of patient-reported trust measures and our hypothesis that NP-led HIV care would demonstrate relational strengths alongside comparable clinical outcomes. Consistent with this framework, we hypothesized that NP-led HIV care would demonstrate comparable clinical outcomes to physician-led care while potentially differing in relational outcomes, particularly patient-provider trust.

Prior studies comparing nurse practitioner and physician HIV care demonstrate comparable quality across key clinical outcomes, including antiretroviral therapy initiation and prescribing, immune markers, viral suppression, and sexually transmitted infection testing.^27^ More recent findings further support the role of nurse practitioners in high-quality HIV care. An analysis of the Centers for Disease Control and Prevention’s Medical Monitoring Project (2019–2021) involving 6,323 adults, found that individuals whose primary HIV care providers were NPs had significantly higher rates of retention in care and were more likely to receive HIV testing recommendations than those with infectious disease (ID) physician providers.^26^ In addition, among young adults, nurse practitioners provided a greater number of HIV prevention and treatment services per patient than both infectious disease and non–infectious disease physicians, including routine HIV care, STI testing, and adherence counseling.^26,27^ Despite this growing evidence base, data specific to adolescents and young adults with HIV remain limited.^28^

We hypothesized that youth receiving NP-delivered HIV care would demonstrate comparable engagement in care and clinical outcomes to those receiving physician-delivered care, while potentially differing in relational outcomes, particularly patient-provider trust. Accordingly, the aim of this study was to compare HIV-specific clinical outcomes and patient-reported outcomes among youth with HIV receiving care from nurse practitioners versus physician providers.

## Methods

This cross-sectional observational study examined clinical characteristics and health outcomes among YWH based on their primary provider type (nurse practitioner [NP] versus physician). Data were abstracted from electronic medical records and included demographics, HIV-specific outcomes, visit types, and EIC. The primary outcome was EIC; secondary outcomes included viral suppression, CD4 measures, visit utilization, patient-provider trust, adherence, mental health measures, and HIV stigma. The reporting of this study conforms to the Strengthening the Reporting of Observational Studies in Epidemiology (STROBE) guideline^30^ (see Supplementary File 1). HIV-specific outcomes included HIV-1 RNA PCR (copies/mL), T-cell CD4 absolute cell counts, and T-cell CD4 percentage. For each participant, viral suppression was assessed using HIV-1 RNA PCR results across the 16-month study period. Participants were classified as suppressed if all HIV RNA PCR results during that period were <200 copies/mL. This binary outcome was used to compare viral suppression across provider types.

Visit types were categorized as in-person, telehealth (video or phone), or hybrid. EIC was operationalized as a binary variable. Participants were classified as “engaged in care” using a modified definition based on Ryan White HIV/AIDS Program retention measures and CDC Medical Monitoring Project criteria. To examine care patterns before and after the onset of the COVID-19 pandemic, the study period included the 8 months before and 8 months after March 15, 2020, the date emergency public health orders were enacted in Colorado. Participants were considered engaged if they had at least one HIV care visit in both time periods, had at least one HIV RNA PCR test during the study window, and demonstrated sustained viral suppression (defined as all HIV RNA PCR results <200 copies/mL).^31,32^ Those not meeting these criteria were classified as “not engaged in care.” Because this composite EIC variable included viral suppression, visit utilization was also examined separately using total HIV care visits, telehealth visits, in-person visits, and hybrid visits.

Patient-reported outcomes were collected using a self-administered survey and included measures of patient-provider trust, ART adherence, and HIV stigma. Trust was assessed using three validated tools: the Healthcare Relationship Trust Scale-Revised (HCR Trust Scale-R),^33^ the Watson Patient Caring Score (WPCS),^34^ and the Patient Trust Assessment Tool (PaTAT).^35,36^ The HCR Trust Scale-R is a 13-item scale measuring perceived provider trust using a 5-point Likert scale, with strong internal consistency (Cronbach’s α = .92) and moderate test-retest reliability (r = .59).^33^ For descriptive categorical analyses, HCR Trust Scale-R scores were grouped according to the distribution of scores in this sample; these categories were not treated as established clinical cut points. The WPCS is a 5-item measure of human caring practices using a 7-point scale (Cronbach’s α = .90).^34^ The PaTAT consists of 25 items that evaluate trust in electronically delivered health services.^35,36^

Adherence to ART was measured using a validated self-report scale widely used in HIV care.^37^ HIV stigma was assessed using the revised 12-item HIV Stigma Scale, which uses a 4-point Likert scale and has demonstrated acceptable internal consistency (Cronbach’s α > .70).^38^ Data collection spanned from June 15, 2019 to November 15, 2020, and was designed to capture eight months before and after the onset of the COVID-19 pandemic, operationalized March 15, 2020, when emergency public health orders were enacted in Colorado. Participants were purposively sampled from two affiliated ambulatory HIV clinics located within a quaternary children’s hospital and its university-based adult counterpart.

Eligible participants were 18 to 30 years of age, living with perinatally or situationally acquired HIV, English-speaking, and receiving or previously received care at one of the two sites. Although the study targeted young adults 18 to 30 years of age, a small number of individuals (n = 7) over the age of 30 years, living with perinatally acquired HIV, were included due to their participation in a long-term observational study and ongoing connection to the adolescent clinical research program. These participants provided additional insights into the transition from pediatric to adult HIV care. We excluded pregnant individuals due to the increased frequency of visits associated with perinatal care standards. This study represents a secondary analysis of data collected as part of a larger observational study for which the sample size was determined based on the parent study objectives.^39^ Recruitment occurred via text, phone, email, and secure messaging through the electronic health record. Surveys were administered via REDCap^40^ at the University of Colorado following electronic informed consent. The Colorado Multiple Institutional Review Board (COMIRB #20-2536) approved the study.

### Statistical Analysis

Statistical analyses were conducted using IBM SPSS Statistics version 27.^41^ Comparisons between nurse practitioner and physician groups were performed using chi-square or Fisher’s exact tests for categorical variables and Wilcoxon rank-sum tests for continuous variables. Adjusted analyses were conducted for key clinical and patient-reported outcomes using models controlling for age, race/ethnicity, gender, education, HIV acquisition mode, and total number of HIV care visits. Total number of HIV care visits was not included as a covariate when total visits or EIC were the outcome of interest.

Survey measures were complete for all participants. One participant with unknown provider type was excluded from provider-level analyses. Most variables had complete data, except for laboratory measures. CD4 outcomes had approximately 14% missingness and HIV RNA PCR measures had approximately 8% missingness. Given the relatively modest level of missing data, analyses were conducted using complete-case data.

## Results

Of the 281 YWH who were screened and approached for participation, 109 individuals (38.8%) provided informed consent, completed the electronic survey, and were included in the final sample for the chart abstraction. Of those not enrolled, 15 (5.3%) declined participation, 121 (43.1%) did not respond to recruitment attempts, and 36 (12.8%) were ineligible. The average age of participants was 26.4 years (SD = 4.04); 59.6% identified as male, 1.8% as transgender female, and 0.9% as transgender male. Regarding race and ethnicity, 37.6% identified as American Indian, Black or African American, or more than one race, and 73.4% identified as non-Hispanic or Latino. A total of 43 participants (39.4%) had perinatally acquired HIV. Seventy participants (64.2%) identified their primary HIV provider as a physician, while 38 (34.9%) received care from a nurse practitioner; one participant had missing provider information and was excluded from provider-level comparisons (Table 1).

**Table 1.** Participant Demographics

Category	Subcategory	Total sample (n = 109) n (%)
Age	Age (mean, SD)	26.4 (4.04)
Age Group	18–24	39 (35.8%)
	25–30	63 (57.8%)
	31 and older	7 (6.4%)
Gender	Female	41 (37.6%)
	Male	65 (59.6%)
	Trans female	2 (1.8%)
	Trans male	1 (0.9%)
Race/Ethnicity^a^	American Indian	3 (2.8%)
	Black or African American	29 (26.6%)
	More than one race	9 (8.3%)
	Unknown	12 (11.0%)
	White or Caucasian	56 (51.4%)
	Hispanic or Latino	29 (26.6%)
	Not Hispanic or Latino	80 (73.4%)
Education	Some high school	11 (10.1%)
	HS graduate or some college	76 (69.7%)
	College degree or higher	22 (20.2%)
HIV Acquisition	Perinatally acquired	43 (39.4%)
	Situationally acquired	66 (60.6%)
Transitioned Care	Pre-transition (still in adolescent care)	37 (33.9%)
	Transitioned to adult care	63 (57.8%)
	In adult clinic, never transitioned	9 (8.3%)
Current HIV Care Provider^b^	Physician	70 (64.2)
	Nurse practitioner	38 (34.9)

^a^Participants chose all races and ethnicities that applied to them.

^b^One participant stated they did not know their provider.

Participant characteristics, including race, ethnicity, gender, and mode of HIV acquisition, did not differ significantly by provider type (Table 2). Viral suppression and EIC were comparable between participants receiving NP and physician care (Table 3). EIC was defined as having at least one pre- and post-COVID visit, one HIV RNA PCR test during the study period, and all HIV RNA PCR results <200 copies/mL. Participants receiving NP care had higher mean CD4 absolute counts (765.0 vs. 613.0 cells/µL; p = 0.043; Cohen’s d = 0.45; 95% CI 0.04-0.89) and CD4 percentages (35.5% vs. 30.8%; p = 0.039; Cohen’s d = 0.47; 95% CI 0.04-0.89) than those receiving physician care (Table 3). After adjustment for age, race/ethnicity, gender, education, HIV acquisition mode, and total HIV care visits, CD4 absolute count remained statistically significant, whereas CD4 percentage was attenuated and became borderline significant. No significant differences were observed in adjusted analyses of viral suppression or EIC (Supplementary Table 2).

**Table 2.** Comparison of Clinical and Visit Characteristics by Provider Type

Variable	Level	Total n(%)	Physician	Nurse practitioner	p-value	Median difference or odds ratio (95% CI)
N^a^	​	109 (100%)	70 (64.2%)	38 (34.9%)	​	​
Gender	Female or trans-female	43 (39.4%)	26 (37.1%)	16 (42.1%)	0.765	1.23(0.51,2.96)
	Male or trans-male	66 (60.6%)	44 (62.9%)	22 (57.9%)	​	​
Race	People of Color and White plus Hispanic	66 (61.1%)	41 (58.6%)	25 (67.6%)	0.483	1.47(0.59,3.76)
	White and non-Hispanic	42 (38.9%)	29 (41.4%)	12 (32.4%)	​	​
Education	Some college or higher	75 (68.8%)	51 (72.9%)	24 (63.2%)	0.409	0.64(0.25,1.63)
	High-school graduate or less	34 (31.2%)	19 (27.1%)	14 (36.8%)	​	​
HIV Mode of Acquisition	Perinatally acquired	43 (39.4%)	29 (41.4%)	14 (36.8%)	0.796	0.83(0.33,2.00)
	Non-perinatally acquired	66 (60.6%)	41 (58.6%)	24 (63.2%)	​	​
Ever Transitioned from Adolescent to Adult Care	Never transitioned to Adult Care	51 (46.8%)	29 (41.4%)	22 (57.9%)	0.151	1.93(0.81,4.69)
	Transitioned to Adult Care	58 (53.2%)	41 (58.6%)	16 (42.1%)	​	​
Telehealth Use^b^	Yes	76 (70.4%)	43 (61.4%)	33 (86.8%)	0.008	4.09(1.35,15.10)
Total HIV Care Visits^c^	Median [IQR]	​	3.00 [2.00, 4.00]	4.00 [3.00, 5.00]	0.019	1.00(0.00,2.00)
Total Telehealth Visits^c^	Median [IQR]	​	0.00 [0.00, 1.00]	1.00 [0.00, 1.00]	0.002	—
Total Hybrid Visits^d^	Median [IQR]	​	0.00 [0.00, 0.00]	0.00 [0.00, 1.00]	0.001	—

Effect estimates are not reported for telehealth and hybrid visit counts because the highly tied distributions produced unstable or clinically uninterpretable median-difference estimates.

^a^Total study sample was 109 participants; however, one individual had missing provider type and was excluded from provider-level comparisons.

^b^Telehealth use is defined as having had at least one telehealth visit during the 16-month study period.

^c^Total HIV care visits, telehealth visits, and hybrid visits represent the number of each visit type per participant over the full study period.

^d^A hybrid visit was defined as an in-person visit in which one or more care team members (e.g., physician, social worker, or case manager) joined remotely via telehealth. Median values of 0.00 indicate that more than half of participants in that provider group did not experience that specific visit type.

**Table 3.** HIV Outcomes, Visits and Engagement by Provider Type

Measure	Level	Nurse practitioner (n = 38)	Physician (n = 70)	p-value	Effect size (95% CI)	Adjusted model p-value
CD4 absolute count (mean, SD)	​	765.0 (365.2)	613.0 (306.1)	0.043	Cohen’s d = 0.451 (0.04,0.89)	0.030
CD4 percentage (mean, SD)	​	35.5 (10.3)	30.8 (10.1)	0.039	Cohen’s d = 0.47 (0.04,0.89)	0.082
HIV RNA PCR <200 copies/mL^a^	Yes	28 (77.8%)	45 (71.4%)	0.636	φ = 0.07 (0.00,1.00)	0.361
Total HIV care visits (median [IQR])	​	4.00 [3.00, 5.00]	3.00 [2.00, 4.00]	0.019	Cohen’s d = 0.46 (0.05,0.85)	0.022
Engaged in care^b^	Yes	17 (44.7%)	34 (48.6%)	0.840	φ = 0.04 (0.00,1.00)	0.892

^a^All HIV RNA PCRs were in this range in the total 16-month study time period.

^b^Engagement in care was defined as having at least one HIV care visit both before and after March 15, 2020 (the COVID-19 emergency declaration in Colorado), at least one HIV RNA PCR test during the study period, and all HIV RNA PCR results <200 copies/mL. Adjusted p-values are from models controlling for age, race/ethnicity, gender, education, HIV acquisition mode, and total number of HIV care visits, except when total visits or engagement in care were the outcome.

Participants receiving NP care had a higher median number of total HIV care visits during the study period than those receiving physician care (4.0 vs. 3.0 visits; p = 0.019; Table 2). Participants receiving NP care were also more likely to have used telehealth during the study period (86.8% vs. 61.4%; p = 0.008; Table 2). When visit modality was examined, a greater proportion of encounters among participants receiving physician care occurred in person (78.0% vs. 22.0%; p = 0.010), whereas a greater proportion of encounters among participants receiving NP care occurred via telehealth (55.0% vs. 45.0%; p = 0.002; data not shown).

Overall, 55 participants (50.9%) had HCR Trust Scale-R scores at or above the sample-derived median which was used for descriptive purposes rather than as a validated clinical cutoff (Table 4). After adjustment, participants receiving NP care remained more likely to have HCR Trust Scale-R scores at or above the sample-derived threshold compared with those receiving physician care (65.8% vs. 42.9%; adjusted p = 0.019). Continuous trust scores did not differ significantly by provider type across the HCR Trust Scale-R, WPCS, or PaTAT.

**Table 4.** Patient-Reported Outcomes by Provider Type

Measure	Level	Nurse practitioner (n = 38)	Physician (n = 70)	p-value	Effect size (95% CI)	Total (n = 108)	Adjusted model p-value
Trust (HCR ≥49)	At or above sample median	25 (65.8%)	30 (42.9%)	0.023	φ = 0.28 (0.09,1.00)	55 (50.9%)	0.019
Trust (HCR 40–48)	Intermediate HCR score	7 (18.4%)	28 (40.0%)	---	---	35 (32.4%)	---
Trust (HCR <40)	Lower HCR score	6 (15.8%)	12 (17.1%)	0.326	φ = 0.15 (0.00,1.00)	18 (16.7%)	0.379
Adherence	Good adherence	33 (86.8%)	51 (72.9%)	0.145	φ = 0.16 (0.00,1.00)	84 (77.8%)	0.202
Depression symptoms (PHQ-4 ≥6)	Depressed	24 (63.2%)	44 (62.9%)	1.000	φ = 0.000 (0.00,1.00)	68 (63.0%)	0.984
Psychological distress (K6 ≥5)	Moderate to severe	14 (36.8%)	22 (31.4%)	0.722	φ = 0.034 (0.00,1.00)	36 (33.3%)	0.631

*Note.* HCR Trust Scale-R categories were created for descriptive purposes based on the distribution of scores in this study sample and are not established clinical cut points. Cutoff values for depressive symptoms on the PHQ-4 and psychological distress on the K6 are based on the cited literature. Adjusted p-values are from models controlling for age, race/ethnicity, gender, education, HIV acquisition mode, and total number of HIV care visits.

No significant differences were observed between provider groups in medication adherence (86.8% for nurse practitioner patients vs. 72.9% for physician patients; p = 0.145), depressive symptoms (PHQ-4 ≥6: 63.2% vs. 62.9%; p = 1.000), psychological distress (K6 ≥5: 36.8% vs. 31.4%; p = 0.722), or HIV stigma (mean difference [95% CI]: 0.02 [−0.23, 0.28]; p = 0.855; Table 4).

## Discussion

The results of this study suggest that nurse practitioners (NPs) and physicians provide comparable HIV care to youth across several critical clinical outcomes, including HIV viral load suppression, EIC, medication adherence, mental health measures, and HIV stigma. Demonstrating comparable outcomes across these domains adds to a growing body of evidence showing that NPs and physicians provide similar care for individuals with chronic health conditions.^42-45^ The adjusted analyses were highly consistent with the unadjusted findings, supporting the overall interpretation that NP and physician care were comparable across key HIV outcomes, with NP care associated with more visits, greater telehealth use, and a higher proportion of above-median HCR Trust Scale-R scores.

Importantly, the diversity of our sample suggests that NPs may also have a role in advancing health equity for youth with HIV. Because most participants identified as Black, Indigenous, People of Color, Hispanic, or members of other historically marginalized groups, these findings should be interpreted within the broader context of structural determinants of health. Workforce shortages, geographic access barriers, insurance limitations, stigma, and racism continue to shape HIV outcomes among youth. Expanding access to NP-delivered HIV care may represent one strategy for addressing these structural barriers and improving equitable access to youth-focused HIV services.

This role is particularly important in the context of a growing HIV provider shortage and persistent geographic disparities in access to care. The NP workforce is more likely to practice in underserved communities and areas with limited healthcare access.^46-49^ Expanding the HIV-trained NP workforce may therefore improve equitable access to specialty HIV care, particularly in rural, underserved, and high-burden regions of the United States.^1,2,15^

Ensuring that NPs provide care that meets the same clinical benchmarks as physicians supports their broader integration into the HIV workforce and offers a sustainable strategy for scaling youth-focused HIV services. Beyond comparable clinical outcomes, NP care was associated with greater telehealth utilization and more frequent contact. These findings suggest that NP-led models may provide greater flexibility in care delivery, a hypothesis that warrants further investigation in longitudinal studies. Importantly, greater telehealth utilization was not associated with reduced laboratory monitoring, with HIV laboratory testing completed by 94.7% of participants receiving NP care and 90.0% of those receiving physician care.

Differences in telehealth utilization should also be interpreted within the context of the COVID-19 pandemic. It is possible that provider-specific roles and responsibilities influenced the adoption of telehealth during this period. For example, some physicians maintained greater inpatient responsibilities, which may have affected patterns of outpatient care delivery. However, both nurse practitioners and physicians provided both in-person and telehealth care throughout the study period.

Participants receiving NP care had higher CD4 absolute counts, while CD4 percentage was higher in unadjusted analyses and borderline significant after adjustment. Immune function is influenced by multiple clinical and social factors, and unmeasured differences in patient complexity may have contributed to these findings despite the absence of a formal provider assignment process, including possibly assigning patients with more compromised immune function to a physician provider. These results warrant further investigation in larger studies.

Trust emerged as an important secondary relational outcome in this study. Because the HCR Trust Scale-R categories were derived from the distribution of scores within this sample rather than validated clinical thresholds, these findings should be interpreted as descriptive and hypothesis-generating. Participants receiving NP care were significantly more likely to have HCR Trust Scale-R scores at or above the sample median. Prior research has linked trust to retention, adherence, and engagement in HIV care, particularly among individuals affected by stigma and marginalization.^28,50^ Trust has also been described by people living with HIV as a central component of high-quality care.^28^

Consistent with the Trust-Link Relational Transition Model,^29^ the higher proportion of NP-treated youth with HCR Trust Scale-R scores at or above the sample median may reflect relational aspects of care that support continuity and engagement. The model proposes that trust develops through ongoing interactions characterized by accessibility, communication, and collaborative relationships. In the current study, participants receiving NP care also reported greater telehealth utilization and more frequent visits, suggesting that increased opportunities for connection may contribute to the development of trust. Although causal relationships cannot be established in this cross-sectional study, these findings support further investigation of trust as a potential mechanism linking provider interactions, healthcare utilization, and HIV-related outcomes among youth.

Rather than being positioned merely as substitutes for physicians, NPs demonstrated comparable clinical outcomes, with observed differences in visit frequency, telehealth utilization, and patient-reported trust that warrant further investigation. Future studies should build on these findings across larger, multi-site samples to further explore how NP-led care may promote health equity and continuity among youth with HIV, especially in underserved or high-need settings.

## Implications for Practice

### Expanding HIV Services Through NP Workforce

Given the high proportion of undiagnosed HIV and low testing rates among young people, findings from this study support targeted investment in developing a nurse practitioner workforce focused on HIV and youth to expand access to testing, treatment, and linkage to care, including through the Ryan White HIV/AIDS Program (RWHAP). Although RWHAP serves more than 500,000 individuals nationally, only one in five patients is a youth with HIV, with similarly low representation across rural and non-rural settings.^51^ Notably, the percentage was roughly equal for rural (20.0%) and non-rural (22.0%) RWHAP providers.^51^ Limited access to youth-focused HIV testing and linkage, particularly in rural and southern regions, has been identified as a contributor to persistent geographic disparities in HIV incidence.^51^ Expanding NP-led HIV services for young people aligns with evidence supporting community- and school-based HIV prevention approaches and may strengthen linkage to care in underserved settings.^52^

Workforce expansion should also be accompanied by appropriate structural supports. Although telehealth and flexible care models may improve access, they may also create challenges for laboratory monitoring and follow-up if systems are not well integrated. Reimbursement policies, telehealth infrastructure, care coordination resources, and mechanisms to ensure timely laboratory testing remain important considerations for sustaining high-quality HIV care.

### School-Based Health Centers as a Strategic Platform

School-based health centers (SBHCs) represent a promising platform for NP-led HIV prevention and linkage to care. A recent CDC initiative implemented across 28 school districts reached approximately two million students and demonstrated the feasibility of integrating HIV testing and referral within school settings.^53^ Nationally, NPs provide the majority of preventive services in SBHCs, positioning them as key providers for youth-focused HIV care.^54^ Evidence from SBHC settings shows improved uptake of STI testing and other preventive services when interventions are delivered by NPs rather than computer-based models,^55^ as well as benefits across reproductive and mental health outcomes.^56^ Given the higher trust and telehealth engagement observed among youth receiving NP care in this study, integrating NP-led relational and telehealth approaches within SBHCs may enhance HIV testing, referral, and continuity of care.

### Community-Based NP Services for Youth With HIV

Beyond school settings, expanding community-based NP HIV services for youth may further improve prevention and engagement. Prior evidence indicates that NPs are more likely than physicians to deliver key HIV prevention services, including pre-exposure prophylaxis prescribing.^27^ Systematic reviews of HIV care delivery models suggest comparable clinical outcomes across provider types, with advanced practice models offering a feasible approach for comprehensive HIV care delivery.^57^ Together with the current findings, this evidence supports the expansion of community-based NP HIV services as a strategy to improve access, trust, and continuity of care for youth with HIV.

High level evidence examining quality, utilization, and cost of NP care compared to physician care found no studies associating NP care with worse outcomes.^42^ A recent study examining harm to patients as a result of full practice authority for NPs showed no evidence of such harm.^58^ On the contrary, several systematic reviews show NPs provide safe, comparable care to physicians.^27,42,43,45,48^ Yet, decision-makers remaining skeptical of the safety of NP care despite the evidence may still advocate for structural supports such as collaborative practice agreements as necessary guards against unintended consequences. This outdated approach to strengthen the healthcare workforce is shown to negatively impact progress toward health equity. Studies show that NPs commute from states with physician oversight to less restrictive states.^59^ In fact, studies suggest NP care improves access to minoritized populations.^60^

### Limitations and Strengths

This study has several limitations. Although this analysis was conducted using data from a larger observational study that was powered for its parent study objectives, this secondary analysis was not specifically powered to detect differences between nurse practitioner and physician care. The sample was relatively small, drawn from a single institution, and participants were not randomly assigned to provider type, which may limit generalizability and introduce residual confounding. Unmeasured differences in patient complexity, illness severity, comorbidities, or provider characteristics may have influenced provider assignment and outcomes despite adjustment for available covariates. In addition, CD4 analyses were conducted post hoc and should be interpreted cautiously. Although adjusted analyses were conducted for key outcomes, the modest sample size limited the number of covariates that could be included and supports cautious interpretation. Despite these limitations, the study provides a valuable foundation for future multi-site investigations of HIV care delivery models for youth with HIV.

Despite these limitations, this study has notable strengths. We assessed multiple patient-reported outcomes using validated instruments, including measures of trust, adherence, and stigma, which are often underexplored in HIV care for adolescents and young adults.^28,38^ Our evaluation of these psychosocial and engagement-related factors in YWH, a population often underrepresented in the literature, provides a unique contribution. Furthermore, our use of patient-reported outcome measures supports the advancement of patient-centered HIV care that includes shared decision-making and individualized care planning.^61^ These elements of care delivery are especially critical to meet the developmental and relational needs of youth living with HIV.^62^

## Conclusion

This study found that nurse practitioners and physicians achieved comparable engagement in care and other key clinical outcomes among adolescents and young adults with HIV. Secondary findings related to patient-provider trust, visit frequency, and telehealth utilization suggest potential relational differences that warrant further investigation. These findings support the role of nurse practitioners as a critical component of a youth-focused HIV workforce at a time of persistent provider shortages and inequities in access to care. The greater proportion of above-median HCR Trust Scale-R scores among youth receiving NP care highlights the importance of relational approaches in sustaining engagement during a vulnerable developmental period. Expanding NP-led HIV care models may represent a feasible strategy to enhance access, continuity, and patient-centered care for youth living with HIV. Future multi-site studies are warranted to further examine how NP-delivered care can be leveraged to advance equity and engagement across diverse HIV care settings.

## Supplemental Material

Supplemental Material - HIV Care for Adolescents and Young Adults: A Cross-Sectional Comparison of Nurse Practitioner and Physician Roles in Engagement in Care, Trust, and Clinical Outcomes Supplemental Material for HIV Care for Adolescents and Young Adults: A Cross-Sectional Comparison of Nurse Practitioner and Physician Roles in Engagement in Care, Trust, and Clinical Outcomes by Emily Anne Barr, Suzanne E. Courtwright, Sheryl Malone-Thomas, Lori Silveira, Deborah Kacanek, Paul Cook, Sean M. Reed, Hillary Dunlevy in Journal of the International Association of Providers of AIDS Care (JIAPAC).

Supplemental Material - HIV Care for Adolescents and Young Adults: A Cross-Sectional Comparison of Nurse Practitioner and Physician Roles in Engagement in Care, Trust, and Clinical Outcomes Supplemental Material for HIV Care for Adolescents and Young Adults: A Cross-Sectional Comparison of Nurse Practitioner and Physician Roles in Engagement in Care, Trust, and Clinical Outcomes by Emily Anne Barr, Suzanne E. Courtwright, Sheryl Malone-Thomas, Lori Silveira, Deborah Kacanek, Paul Cook, Sean M. Reed, Hillary Dunlevy in Journal of the International Association of Providers of AIDS Care (JIAPAC).
