# Supplementary material for: HIV Care for Adolescents and Young Adults: A Cross-Sectional Comparison of Nurse Practitioner and Physician Roles in Engagement in Care, Trust, and Clinical Outcomes
Source: J Int Assoc Provid AIDS Care. 2026 Oct 1;25:23259582261485418. doi: 10.1177/23259582261485418 (PMC13735709; doi:10.1177/23259582261485418)
Supplement: Supplementary file 1 [file sj-pdf-1-jia-10.1177_23259582261485418.pdf]

STROBE Statement—Checklist of Items That Should Be Included in Reports of Cross-Sectional Studies

Supplementary File 2

Manuscript: HIV Care for Adolescents and Young Adults: A Cross-Sectional Comparison of Nurse Practitioner and Physician Roles in Engagement in Care, Trust, and Clinical Outcomes

Section/topic	Item	Recommendation	Reported on page(s)
**Title and abstract**	1(a)	Indicate the study's design with a commonly used term in the title or the abstract	1
	1(b)	Provide in the abstract an informative and balanced summary of what was done and what was found	1
**Background/rationale**	2	Explain the scientific background and rationale for the investigation being reported	3–6
**Objectives**	3	State specific objectives, including any prespecified hypotheses	6
**Study design**	4	Present key elements of study design early in the paper	1, 7
**Setting**	5	Describe the setting, locations, and relevant dates, including periods of recruitment, exposure, follow-up, and data collection	8–9
**Participants**	6(a)	Give the eligibility criteria, and the sources and methods of selection of participants	9
**Variables**	7	Clearly define all outcomes, exposures, predictors, potential confounders, and effect modifiers. Give diagnostic criteria, if applicable	7–8
**Data sources/measurement**	8	For each variable of interest, give sources of data and details of methods of assessment (measurement). Describe comparability of assessment methods if there is more than one group	7–9
**Bias**	9	Describe any efforts to address potential sources of bias	8–10, 17
**Study size**	10	Explain how the study size was arrived at	9, 17
**Quantitative variables**	11	Explain how quantitative variables were handled in the analyses. If applicable, describe which groupings were chosen and why	7–8
**Statistical methods**	12(a)	Describe all statistical methods, including those used to control for confounding	8–10

Section/topic	Item	Recommendation	Reported on page(s)
	12(b)	Describe any methods used to examine subgroups and interactions	Not applicable; no subgroup or interaction analyses reported
	12(c)	Explain how missing data were addressed	10
	12(d)	If applicable, describe analytical methods taking account of sampling strategy	Not applicable; no complex sampling strategy used
	12(e)	Describe any sensitivity analyses	Not applicable; no sensitivity analyses reported
**Participants**	13(a)	Report numbers of individuals at each stage of the study—for example, numbers potentially eligible, examined for eligibility, confirmed eligible, included in the study, completing follow-up, and analysed	10
	13(b)	Give reasons for non-participation at each stage	10
	13(c)	Consider use of a flow diagram	10 (numbers and reasons reported in text; no flow diagram)
**Descriptive data**	14(a)	Give characteristics of study participants (for example, demographic, clinical, social) and information on exposures and potential confounders	10; Tables 1–2 cited in manuscript
	14(b)	Indicate number of participants with missing data for each variable of interest	10
**Outcome data**	15	Report numbers of outcome events or summary measures	10–11; Tables 3–4 cited in manuscript
**Main results**	16(a)	Give unadjusted estimates and, if applicable, confounder-adjusted estimates and their precision (for example, 95% confidence interval). Make clear which confounders were adjusted for and why they were included	10–11
	16(b)	Report category boundaries when continuous variables were categorized	8, 11
	16(c)	If relevant, consider translating estimates of relative risk into absolute risk for a meaningful time period	Not applicable

Section/topic	Item	Recommendation	Reported on page(s)
**Other analyses**	17	Report other analyses done—for example, analyses of subgroups and interactions, and sensitivity analyses	11 (adjusted and secondary analyses); no subgroup, interaction, or sensitivity analyses
**Key results**	18	Summarize key results with reference to study objectives	12
**Limitations**	19	Discuss limitations of the study, taking into account sources of potential bias or imprecision. Discuss both direction and magnitude of any potential bias	13–14, 17
**Interpretation**	20	Give a cautious overall interpretation of results considering objectives, limitations, multiplicity of analyses, results from similar studies, and other relevant evidence	12–18
**Generalisability**	21	Discuss the generalisability (external validity) of the study results	17
**Funding**	22	Give the source of funding and the role of the funders for the present study and, if applicable, for the original study on which the present article is based	This is attached to the unblinded title page.

Page-number note: Locations refer to the numbered manuscript pages in the revised tracked blinded version dated August 6, 2026. The manuscript narrative ends on page 18 and the AMA reference list begins on page 19.

Checklist source: STROBE Statement checklist for cross-sectional studies (www.strobe-statement.org).
