# Supplementary material for: HIV Care for Adolescents and Young Adults: A Cross-Sectional Comparison of Nurse Practitioner and Physician Roles in Engagement in Care, Trust, and Clinical Outcomes
Source: J Int Assoc Provid AIDS Care. 2026 Oct 1;25:23259582261485418. doi: 10.1177/23259582261485418 (PMC13735709; doi:10.1177/23259582261485418)
Supplement: Supplementary file 2 [file sj-pdf-2-jia-10.1177_23259582261485418.pdf]

Supplemental Table 1. Lab Values

Lab Value	Everyone (N=109)
HIV RNA PCR copies/mL	*M* 50707.41<br>*SD* 570907.21<br>Median 20.00<br>Mode 0.00<br>Min 0.00<br>Max 10000000.00
HIVPCR Tests	*M* = 3.12<br>*SD* = 2.18<br>Median = 3.00<br>Mode = 2.00<br>Min = 0<br>Max = 13
HIVPCR % of detectable tests	*M* = 27.05<br>*SD* = 34.48<br>Median = 16.03<br>Mode = 0.00<br>Min = 0.00<br>Max = 100.00<br>IQR = [0.00, 50.00]<br>0% *n* = 49, 45.0%<br>100% *n* = 12, 11.0%
CD4 absolute	*M* = 666.11<br>*SD* = 336.43<br>Median = 605.00<br>Mode = 408.00<br>Min = 126.00<br>Max = 1726.50
CD4%	*M* = 32.53<br>*SD* = 10.02<br>Median = 32.13<br>Mode = 30.00<br>Min = 10.00<br>Max = 58.00

Note: The statistics here reflect the *M*, *SD*, etc., of the computed mean of CD4 absolute count and CD4% across all visits for each person, rather than descriptives across all individual tests. However, HIV RNA PCR tests were reported as individual tests in order to indicate an outlier of a participant with > 10,000,000 copies.
